# Clinical trial allocation in multinational pharmaceutical companies – a qualitative study on influential factors

**DOI:** 10.1002/prp2.317

**Published:** 2017-05-19

**Authors:** Tilde Dombernowsky, Merete Haedersdal, Ulrik Lassen, Simon F. Thomsen

**Affiliations:** ^1^ Department of DermatologyCopenhagen University Hospital BispebjergBispebjerg Bakke 23, entrance 9DK‐2400Copenhagen NVDenmark; ^2^ Department of OncologyCopenhagen University Hospital RigshospitaletBlegdamsvej 9, entrance 5073DK‐2100Copenhagen ØDenmark; ^3^ Department of Biomedical SciencesUniversity of CopenhagenBlegdamsvej 3bDK‐2200Copenhagen NDenmark

**Keywords:** Clinical trial allocation, patient recruitment, pharmaceutical companies, qualitative research, trial site selection

## Abstract

Clinical trial allocation in multinational pharmaceutical companies includes country selection and site selection. With emphasis on site selection, the overall aim of this study was to examine which factors pharmaceutical companies value most when allocating clinical trials. The specific aims were (1) to identify key decision makers during country and site selection, respectively, (2) to evaluate by which parameters subsidiaries are primarily assessed by headquarters with regard to conducting clinical trials, and (3) to evaluate which site‐related qualities companies value most when selecting trial sites. Eleven semistructured interviews were conducted among employees engaged in trial allocation at 11 pharmaceutical companies. The interviews were analyzed by deductive content analysis, which included coding of data to a categorization matrix containing categories of site‐related qualities. The results suggest that headquarters and regional departments are key decision makers during country selection, whereas subsidiaries decide on site selection. Study participants argued that headquarters primarily value timely patient recruitment and quality of data when assessing subsidiaries. The site‐related qualities most commonly emphasized during interviews were study population availability, timely patient recruitment, resources at the site, and site personnel's interest and commitment. Costs of running the trials were described as less important. Site personnel experience in conducting trials was described as valuable but not imperative. In conclusion, multinational pharmaceutical companies consider recruitment‐related factors as crucial when allocating clinical trials. Quality of data and site personnel's interest and commitment are also essential, whereas costs seem less important. While valued, site personnel experience in conducting clinical trials is not imperative.

## Introduction

Clinical trial allocation in multinational pharmaceutical companies is a complex process determined by multiple factors. The process contains two fundamental steps. First, clinical trials are allocated to different geographic regions during a country selection process led by the headquarters of the company. Subsequently, a site selection process is conducted during which the subsidiaries of the regions involved contact potential trial sites and make an evaluation of these sites. Based on this evaluation, the company decides which trial sites it prefers to collaborate with. Country selection depends on factors such as patient availability, national treatment practices, and sales potential of the drug, but also on the performance of subsidiaries and trial sites in prior trials. Therefore, the subsidiaries of a company are to some extent internally competing to attract clinical trials to their region.

Many national governments and trial sites are interested in conducting industry‐sponsored clinical trials, as these are often considered beneficial for the patients included, the trial sites involved, and the country as a whole. However, knowledge of which factors pharmaceutical companies values most when allocating clinical trials is sparse. To our knowledge, only one published study has investigated factors that influence trial allocation in Europe (Gehring et al. 2013). A better understanding of the pharmaceutical industries' allocation of clinical trials is essential if governments and trial sites are to attract and retain more industry‐sponsored clinical trials.

Accordingly, with emphasis on site selection, the overall aim of this study is to examine which factors pharmaceutical companies value most when allocating clinical trials. The specific aims are (1) to identify which departments internally in pharmaceutical companies, and possibly externally, are key decision makers during country and site selection, respectively, (2) to evaluate by which parameters subsidiaries are primarily assessed by headquarters with regard to conducting clinical trials, and (3) to evaluate which site‐related qualities companies value most when selecting trial sites.

## Materials and Methods

### Design, setting, and participants

Eleven semistructured interviews were conducted among employees engaged in clinical trial allocation at 11 multinational pharmaceutical companies (Appendix 1).

The following inclusion criteria were set:

1. The participants should be working with allocation of clinical trials at a multinational pharmaceutical company.
2. The company should be a multinational pharmaceutical company within the top 25 in terms of economic turnover (PM Live 2016).
3. The company should have a Danish subsidiary running clinical trials in Denmark.

We recruited the participants through the Danish Association of the Pharmaceutical Industry (LIF). Participants were included consecutively. In total, 16 eligible participants were contacted via an email describing the aim of the project. Five of these declined to participate. Ten of the included participants were employed at the Danish subsidiary of their company at the clinical operations department, whereas one was employed at the European regional clinical operations department. All participants had at least 5 years of experience with clinical trial allocation. The strategic and operational work patterns within the companies were unknown to the authors. Likewise, the participants' role in the company and perceptions regarding trial allocation were unknown.

### Data collection

From 31st March to 13th May 2016, 11 semistructured in‐depth interviews were conducted in Danish using an interview guide (Appendix 2 (English), Appendix 3 (Danish)). The first part of the interviews was focused on the company's internal organization with emphasis on decision makers during trial allocation. The second part was focused on the site selection process, emphasizing site‐related qualities. The participants were encouraged to speak freely, and in relation to the semistructured style of the interview, the interview guide was not followed strictly but served as a guiding tool. The guide was modified after the first and fourth interview. The interview guide and the citations presented were translated by a native speaker of English.

Seven of the interviews were conducted face‐to‐face at the companies' Danish subsidiaries, whereas four of the interviews, for practical reasons, were conducted by telephone. The interviews lasted between 1 h and 15 min and 1 h and 45 min and were conducted, transcribed, and analyzed by the first author (TD). The seven face‐to‐face interviews were digitally, audio‐recorded. Interview passages concerning the evaluation of trial sites were transcribed verbatim, whereas the rest was transcribed nonverbatim. The four telephone interviews were not audio‐recorded. During the interviews, as many notes as possible were made by the interviewer (TD). The text was elaborated on and corrected in relation to the specific questions asked immediately after the interviews. Subsequently, the participants were contacted by email or phone to elaborate on their answers, if necessary. These responses were included in the data analysis. By Danish law, ethics approval was not required for this study.

### Data analysis

**Figure 1:**
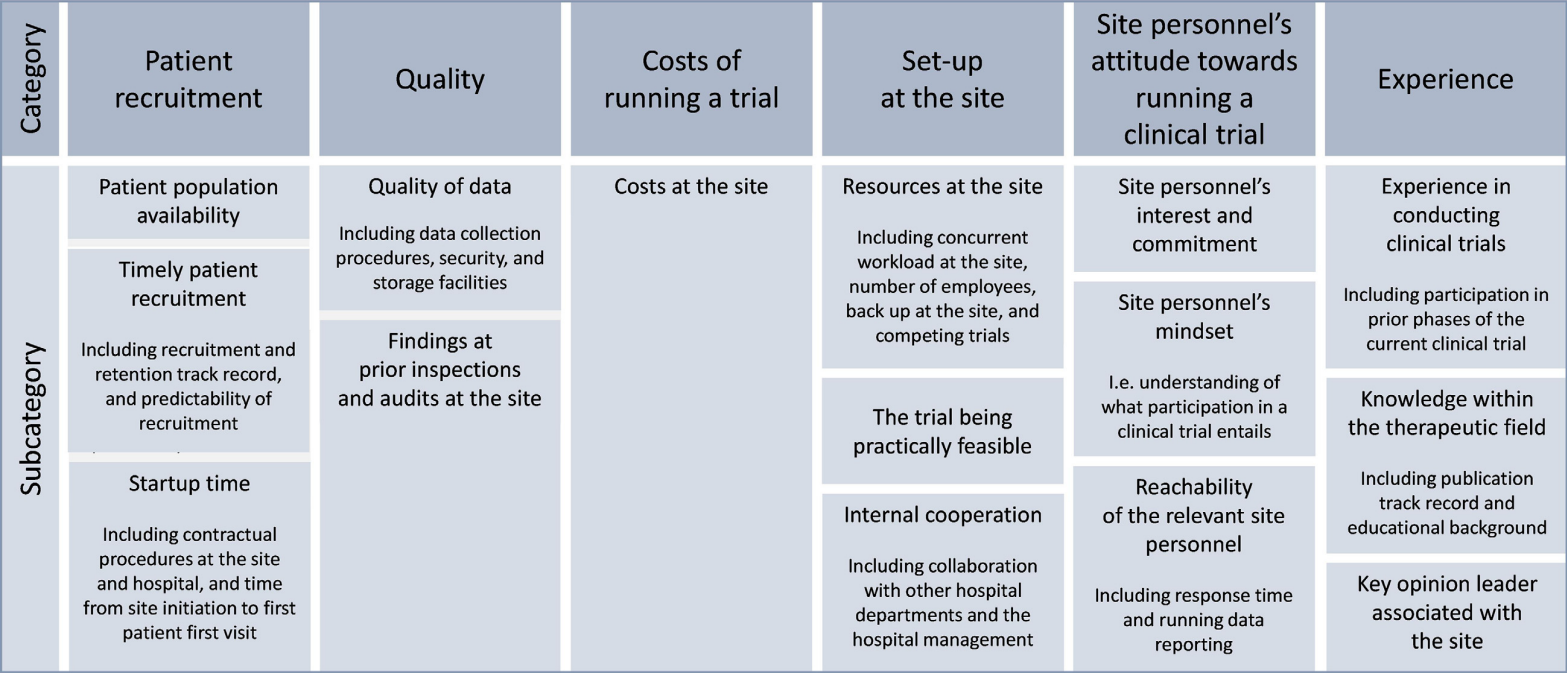
Categorization matrix containing categories of site‐related qualities by which pharmaceutical companies theoretically evaluate clinical trial sites during site selection.

The interviews were analyzed using deductive content analysis as described by Elo and Kyngäs (2008). We decided to analyze only the manifest content of the interviews (Graneheim and Lundman 2004). A categorization matrix was created before the interviews were conducted. It contained 6 categories and 14 subcategories of site‐related qualities by which pharmaceutical companies theoretically evaluate trial sites during site selection. The categories were modified from categories made by Gehring et al. (2013) and Harper and Zuckerman (2006). One subcategory *(site personnel's mindset)* was added during the data analysis (Fig. 1).

The data analysis contained three main phases:

#### Overview

The transcripts were read several times to obtain an overall impression of the content.

#### Analysis of site‐related qualities

**Figure 2:**
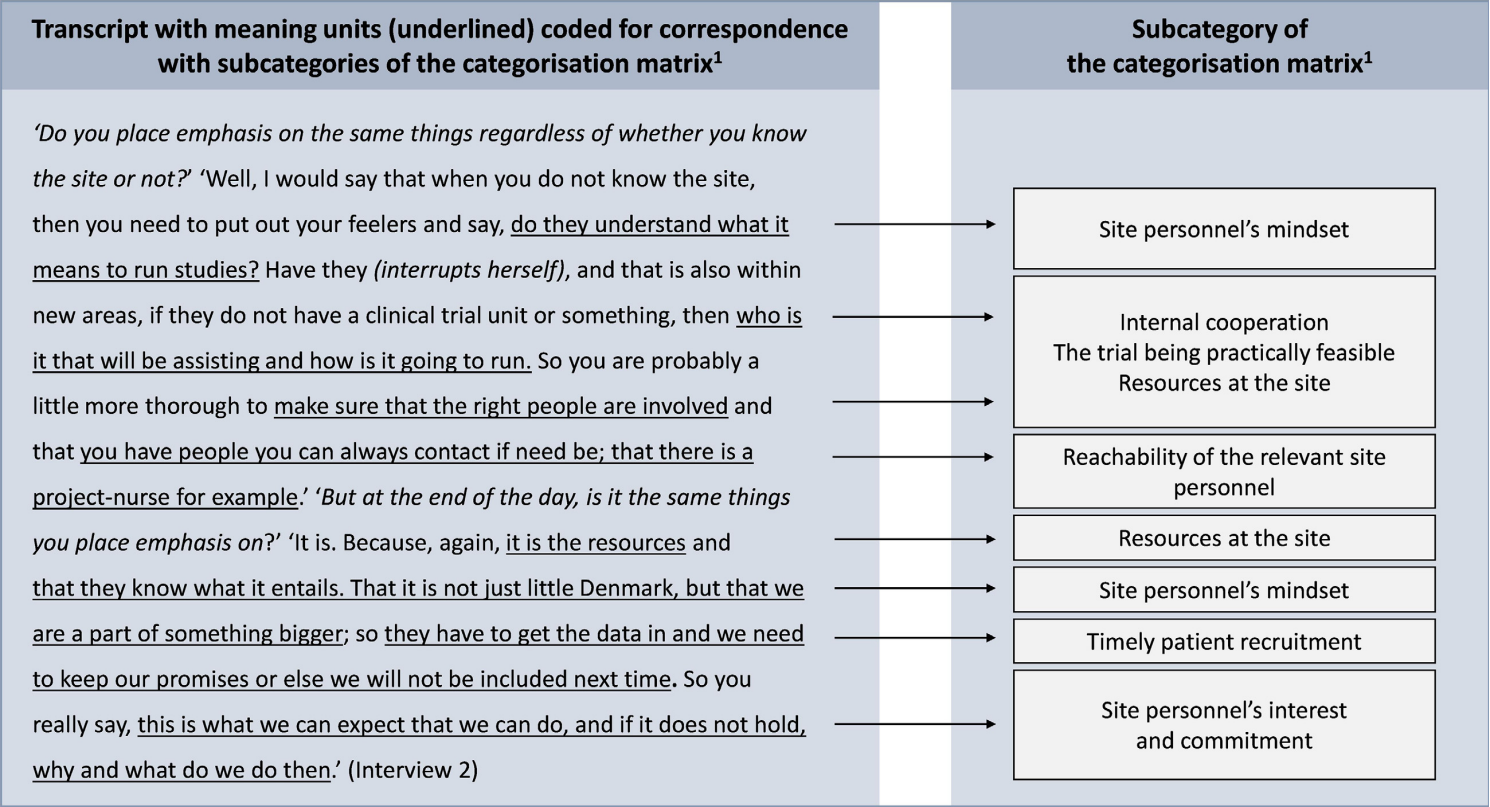
An example of coding of data to the categorization matrix^1^: ^1^The categorization matrix is displayed in Figure 1.

The categorization matrix was used as a lens during the individual reading of the transcripts. Meaning units were defined in reference to Graneheim and Lundman (2004) and were interpreted with regard to the context of the specific interview. The meaning units were color coded in relation to the corresponding subcategory of the categorization matrix. Subsequently, all meaning units that did not fit the categorization matrix were interpreted together in an inductive manner to examine if any possible categories were overlooked. The subcategory *site personnel's mindset* was added to the categorization matrix; eight participants emphasized site‐related qualities that could be summarized by this subcategory exclusively. Finally, statements related to each subcategory were reviewed together across all interviews in order to evaluate each subcategory independently. The coding of data is exemplified in Figure 2.

#### Analysis of the remaining content

The remaining content was interpreted without coding; however, the interviews were still systematically reviewed to ensure that all relevant statements were included.

## Results

### Decision makers during clinical trial allocation

The key decision maker during country selection was the headquarters, in some of the companies in collaboration with regional departments. The subsidiaries were not decision makers in this process, but the participants stated that the subsidiaries influence country selection by different means such as giving feedback to the headquarters in feasibility questionnaires, introducing the headquarters to potential trial sites, and aiming for key opinion leaders at advisory boards. During site selection, the key decision makers were the subsidiaries. In most of the companies, the subsidiaries solely decided which trial sites to include, although the headquarters formally had to approve the selected sites. Nine companies outsourced clinical trials to clinical research organizations (CROs). In fully outsourced clinical trials, the CROs were decision makers during country selection in collaboration with the headquarters and regional departments. Generally, the CROs and the subsidiaries collaborated on deciding which trial sites to include during site selection.

### Assessment of the subsidiaries

All of the participants believed that timely patient recruitment is the most or one of the most important factors by which the headquarters evaluate the subsidiaries. Seven participants also mentioned quality of data as essential. However, two participants disagreed that the headquarters highly values quality. All participants expressed that costs of running a clinical trial are not as important to the headquarters as other factors.

It was emphasized that the Danish/Nordic subsidiaries have to keep demonstrating a successful patient recruitment to ensure allocation of future trials to the country; small countries are not automatically assigned trials and they risk being deselected if patient recruitment is insufficient. In contrast, some argued that large countries are often selected regardless of their prior recruitment performance due to other factors such as sales potential of the drug.

Provided that the data are ok, that the quality is sufficient, then that is what we are assessed by; do we deliver what we promised to deliver by the agreed upon deadline. If we do so, we can be sure to be offered lots of trials (…) We are also assessed on whether we make database logs, queries, etc. on time, but overall, the graphs we are presented for are regarding recruitment. That is why I say that I want to know if my sites can deliver the number of patients they promise, because it matters at the other end.' (Interview 1).

If we have the patients, then if it ends up costing 100,000 more in total or whatever it might be, it is not what they are looking at. The U.S. is already more expensive but they are still running trials. So that is not the most important thing, so to speak.' (Interview 2).

### Evaluation of site‐related qualities

Overall, the site‐related qualities most commonly emphasized during interviews were patient population availability, timely patient recruitment, resources at the site, and site personnel's interest and commitment (Fig. 1). These were emphasized more than, for example, experience, quality of data, and costs. Study participants argued that site selection is based on a weighing of benefits and disadvantages in each case, taking also into account the number of sites available. Furthermore, the qualities that are the most important vary by type of protocol, phase, and therapeutic area. Two participants noted that when a trial demands both blinded and unblinded personnel, a high level of resources at the site is required. Another explained that experience within the therapeutic field is often essential in an early phase II trial, as the investigator has to be good at distinguishing side effects from symptoms of the disease.

#### Patient recruitment

All of the participants spontaneously mentioned that patient population availability and timely patient recruitment are crucial when selecting trial sites. These qualities were repeatedly emphasized. Study participants all described how they aim to get a valid estimation of the number of patients the site personnel believe to be able to recruit, as this is usually crucial to a successful patient recruitment and therefore a high priority to the company. The importance of a rapid startup time was also emphasized.

It is a lot of the same things [we emphasize] as when we select the countries. It is how many patients we can recruit per site per month. And within that discussion we also cover how quickly we can begin, because the more months they have available for enrolment, the better the chance is that they will reach their target in regard to the number of patients they committed to. So those are the most important factors. (Interview 5).

It is first and foremost the quality as well as their ability to prove that they can deliver the patients; that they have really looked at their database and can tell us whether they have the patients and that they are quick; that they already have them lined up before we start so that they can take a patient as soon as we are ready.' ‘*So what I am hearing you say is that the startup phase matters*?’ ‘Yes, it matters a lot. And of course that they deliver the number of patients they promised. That also means everything.’ (Interview 3).

#### Quality

Only half of the participants spontaneously mentioned quality‐related aspects. However, when asked, the participants stated that they find a high level of quality indispensable. One said that they would never compromise on quality, and another argued that she would rather have data of high quality from a few patients than data of low quality from a large number of patients, as some might have to be removed from the dataset subsequently. It was stated that if there have been findings at prior audits or inspections at a site, the company will usually address this by closely monitoring and supporting the site in future trials.

We really work a lot on the quality, and it is our job to go out and help to ensure that the quality is acceptable, but if we have sites that fundamentally do not understand their responsibility ‐ that the data and patient security are paramount ‐ then that makes our job very difficult and in the end, we have to let a site like that go.(Interview 6).

#### Costs

Generally, costs at the trial site were described as less important than other factors. Eight participants conceded that the headquarters usually approve the selected sites regardless of the costs at the site. However, two participants argued that a rise in costs at Danish sites could be problematic, as the headquarters consequently might choose to allocate fewer trials to the country.

*When you are at a site, how much does the price mean to you, when you are evaluating them prior to a possible collaboration?* Well I would say that as long as we can *(interrupts herself)*. The price just needs to be within what is OK from a compliance point of view. It should not be so high that we can get accused of overpaying, so we have a grand plan that we work around. I think most within the industry use these grand plans (…) *Have you experienced that Global [the headquarters] challenges the price that you have on a site? Have you experienced that*? Only if it is over what the grand plan says, and then I am asked to justify why the price is acceptable. *If you do, do you usually get an OK?* Yes. Yes, if you can provide arguments for it. (Interview 8).

#### Set up at the site

All participants mentioned that they thoroughly evaluate resources at the site as these influence patient recruitment during a trial considerably. Five participants argued that they find it important to talk to the operational site personnel (study coordinators, study nurses, etc.) as they, in contrast to the investigator, know if the study is feasible and the resources available.

‘And I would say that the second most important factor is that they have the resources it requires; the employee‐related resources they have on the site. That they have enough to complete the study within the timelines we have set up. And I would say that all the more practical things concerning equipment, room facilities, storing of drugs, those are more formalities. They are never a problem.’ (Interview 5).

#### Site personnel's attitude toward running a clinical trial

The importance of a high level of interest and commitment among site personnel were emphasized repeatedly. Further, all participants confirmed that they find this quality crucial for a trial to succeed. One participant stressed that site personnel cannot convince patients to participate in a trial if they are not enthusiastic about the trial themselves. The need for site personnel to have the right mindset was also emphasized. It was stated that site personnel need to ‘know what the role of being principal investigator entails’ (Interview 1), ‘not be dismissive of documentation requirements’ (Interview 3), and ‘understand how time‐consuming it is’ (Interview 11).

The interest and the resources are very important. We put a lot of effort into aligning expectations. It is, after all, a collaboration. Both partners have to find it interesting (…) We have a thorough dialogue with the site about what it is about and what our expectations are. Is this really something that the site wants to participate in? (Interview 9).

#### Experience

Site personnel experience in conducting clinical trials was referred to as valuable but not imperative. Seven participants argued that a lack of experience does not exclude a site from participation in clinical trials as the company can compensate by allocating more resources to training and monitoring at the site. However, it was also argued that experience is very important in early phase trials as these contain a circumstantial number of procedures that need to be completed within a narrow time limit; limited experience is more acceptable in phase III and IV clinical trials. Depending on the kind of trial and whether the subsidiary has got the resources, the company will select or deselect inexperienced sites.

‘*If you have a new site that is untrained, is that something that will exclude them from participation?*’ ‘No. No, it is not. We just would not include only brand new sites for a clinical trial. But we would really like to expand to have, for example, one or two or whatever would be possible, because it requires significantly more resources. Because they need training. That is the issue. That that is also the case for them. It is also on their side, right?’ ‘*But what I am hearing you say is that it is not like it is out of the question that they can participate*?’ ‘No, no not at all. On the contrary, we really want to expand too, because it is also a way to expand our research in Denmark and access to the patients.’ (Interview 9).

Having a key opinion leader associated with the site was not highly valued. Many argued that the subsidiaries do not include a site because of a specific key opinion leader if this can compromise patient recruitment. However, all participants conceded that they sometimes experience disagreement between the marketing department or the headquarters and the clinical operations department as whether or not to include a site because of an important key opinion leader. Some argued that key opinion leaders presumably play a greater role in the large countries.

## Discussion

The main findings of this study suggest that recruitment‐related factors and quality of data are essential to multinational pharmaceutical companies when allocating clinical trials, whereas costs of running trials seem less important. Furthermore, site personnel's interest and commitment are apparently imperative, whereas experience in conducting clinical trials is not.

Patient population availability and timely patient recruitment at a site were described as crucial to pharmaceutical companies regardless of the type of clinical trial. In addition, all study participants believed that the headquarters of their company find timely patient recruitment essential when evaluating the subsidiaries. It is expected that recruitment‐related factors are among the most important factors during trial allocation, as a successful recruitment is crucial to a successful clinical trial. Furthermore, recruitment is often one of the most challenging parts of running a trial, and in nearly 80% of clinical trials, enrolment timelines are not met (Kremidas 2011). Moreover, pharmaceutical companies have only limited influence on the recruitment once a trial is running; they basically depend on site personnel recruiting the patients. In contrast, it is easier for the companies to influence other factors. As the participants of this study expressed, the companies can somewhat ensure sufficient data quality and compensate for a lack of experience among site personnel by allocating extra resources to monitoring and training at the site.

The key decision maker during country selection is the headquarters. Therefore, trial sites indirectly influence country selection as they basically control patient recruitment, which is highly valued by the headquarters. Site personnel should be aware of this and aim to gain a successful recruitment not just for the benefit of the trial site but for the benefit of the country as a whole. However, it is plausible that the indispensability of timely patient recruitment primarily applies to small countries, as large countries may benefit from other factors. This is supported by the fact that the United States is involved in numerous clinical trials despite investigators in the United States enrolling only two‐thirds as many subjects as investigators in the rest of the world (English et al. 2010).

The costs of running clinical trials seemed less important than other factors during both country and site selection. This corresponds to the findings by Industry Standard Reports in 2009. They found that among 362 clinical trials stakeholders, 80% preferred to reach enrolment goals 10% more quickly, rather than cutting costs by 20% (Gossen 2011). The questionnaire study made by Gehring et al. (2013) also found cost factors to be less important than other factors during both country and site selection. Respondents were asked to divide 100 points across four different factors impacting trial site selection (investigator, hospital/unit, environmental, and cost factors). Among the 341 clinical trials stakeholders who responded, cost factors were generally rated the lowest number of points. Moreover, costs of running trials were significantly less important than the pool of eligible patients in the region, speed of approvals, and presence of disease management networks (Gehring et al. 2013).

Data from this study indicate that inexperienced trial sites should not feel excluded from engaging in clinical trials. Seemingly, a trial site having enthusiastic and committed site personnel will be selected for phase III and IV trials despite a lack of experience, if the subsidiary has the resources to support the site sufficiently. Generally, pharmaceutical companies seem to highly value the right mindset and interest and commitment among site personnel when selecting trial sites. Hospital managements and trial sites that wish to attract more industry‐sponsored trials might benefit from focusing more on these nonmeasurable qualities. Firstly, it seems beneficial to have easily reachable site personnel and a quick response time when communicating with the companies. Secondly, before committing to a trial, hospital management teams and investigators should prioritize consulting the operational site personnel to assess their immediate attitude toward running the trial. Moreover, it might be beneficial to include nonmeasurable qualities such as reachability and commitment in advertising material alongside traditional factors such as recruitment rates in prior trials.

### Strengths and limitations

All interviews were conducted and transcribed by the same researcher. We believe that this is a strength, as the interviews in this manner were conducted in a uniform matter and the interpretation was aligned. Only one author made the data analysis. However, we believe that this is not a considerable limitation as a stringent data analysis was used, and the interviews did not require interpretation with a high level of abstraction. The study has other noteworthy limitations though. Most importantly, data saturation was not met as it was difficult to comprehensively cover the study aims. Secondly, the participants constituted a homogenous group. Thus, the gained information is limited to this group of clinical trial stakeholders. One might argue that the results are only representative for Danish subsidiaries. However, all participants believed that the subsidiaries of different countries basically evaluate trial sites in the same way, including the same parameters.

## Conclusions and future studies

This study found that multinational pharmaceutical companies consider recruitment‐related factors as crucial when allocating clinical trials. Quality of data and site personnel's interest and commitment are also essential, whereas costs of running the trials seem less important. While valued, site personnel experience in conducting clinical trials is not imperative.

Numerous aspects of this area are still incompletely understood. Future studies should further investigate what influences trial site selection, including how the importance of each site‐related quality varies by type of protocol, trial phase, and therapeutic area. In addition, it would be interesting to examine potential differences in site selection between countries. Moreover, knowledge of which factors pharmaceutical companies emphasize when allocating clinical trials to specific countries is still sparse. Finally, it would be relevant to examine the role of CROs during clinical trial allocation.

## Author Contribution

All authors were involved in the design of the study and all authors contributed to the intellectual content of the manuscript. TD collected the data and developed the first draft of the manuscript. MH, UL, and SF contributed to the critical revision of the data and results, and revised the manuscript. The final version of the manuscript is approved by all authors. TD is the guarantor.

## Disclosures

All authors have completed the ICMJE uniform disclosure form at www.icmje.org/coi_disclosure.pdf (available on request from the corresponding author) and declare: no support from any organization for the submitted work other than those mentioned in the funding statement; no other relationships or activities that could appear to have influenced the submitted work.

## Appendix 1 List of the multinational pharmaceutical companies at which the 11 participants of the study were employed

AbbVie

AstraZeneca

Biogen

Bristol‐Myers Squibb

Eli Lilly

Merck Sharp & Dohme (MSD)

Novartis

Novo Nordisk

Pfizer

Roche

Sanofi

## Appendix 2 Interview guide in English

### Part 1

*The first questions attempt to identify key decision‐makers during allocation of clinical trials that your company sponsors. The aim is to examine which departments internally in the company, and possibly externally, are decision‐makers during allocation of clinical trials on a country and site level. Moreover, the aim is to examine if decision‐makers differ depending on which trial phase and therapeutic area the clinical trials concern*.

Would you please start by telling a little bit about who makes the decisions when your company allocates clinical trials?

Can you elaborate on which role the subsidiaries and headquarters respectively play during the allocation process?

Does who makes the country‐ and site selection differ depending on the kind of clinical trial?

Is the allocation process generally the same regardless of the specific phase and therapeutic area?

By which parameters does your headquarters assess the subsidiaries before and after conducting a clinical trial?

Does your company sometimes outsource decisions regarding country and site selection to others ‐ for example CROs?

When outsourcing clinical trials, is it typically clinical trials within certain phases or therapeutic areas that are being outsourced?

When outsourcing, is it both country selection and site selection that are being outsourced?

### Part 2

*The following questions address how your company evaluates a trial site prior to a potential collaboration including which factors are essential to whether or not your company chooses to engage in such a partnership. The aims are to explore which parameters your company values most when evaluating potential trial sites and on which basis the evaluation is done*.

Does the company have a decision model or unified guidelines which it uses during site selection?

If yes: Does the company have unified guidelines for the different subsidiaries to use?

If no: On what basis is site selection then done?

Do you experience the site selection process as similar from case to case or as somewhat more unstructured?

Is the site selection process the same regardless of trial phase and therapeutic area?

What do you and your colleagues take into account when assessing the benefits and drawbacks of a clinical trial site?

Can you give an example of parameters that are essential for some types of clinical trials, but not essential for other types of clinical trials?

Can you elaborate on which parameters you value the most?

Are there certain circumstances or parameters which are almost always important?

If you are assessing a site which your company has not cooperated with before, which parameters do you particularly value?

What would you prefer that the sites you cooperate with were better at?

How often does it happen that a site with which your company wishes to cooperate on a clinical trial declines the offer?

Are data regarding the patient recruitment rate in prior trials important to you when assessing a site? Do you systematically ask about this information during the feasibility process or at the site selection visit?

When performing site selection, do you always ask about deviations at prior inspections at the site?

How often do you think your company chooses to cooperate with a trial site that does not perform satisfactorily because you have to take specific organizations or persons such as key opinion leaders into account?

Do you think that the things we have discussed apply to the company as a whole or only to your department?

Do you think that other subsidiaries at your company evaluate trial sites in the same way as you, including the same parameters?

Is there anything else you would like to add?

## Appendix 3 Interview guide in Danish

### Del 1

*De første spørgsmål handler om, hvem der tager beslutninger omkring placeringen af de kliniske forsøg, som din virksomhed er sponsor for. Formålet er at belyse, hvem der internt i virksomheden og evt. eksternt står for placeringen af virksomhedens kliniske forsøg på landebasis og på sitebasis. Derudover er formålet at belyse, om der er forskel på, hvem der står for placeringen af de kliniske forsøg, alt efter hvilken fase og terapeutisk område, der er tale om*.

Vil du starte med at fortælle lidt om, hvem der tager beslutningerne omkring placeringen af kliniske forsøg i din virksomhed?

Kan du uddybe, hvilken rolle henholdsvis datterselskaberne og hovedkvarteret har ved placeringen af forsøg?

Er det forskelligt, hvem der udfører lande‐ og site selektion, afhængig af hvilken type forsøg der er tale om?

Er processen ved tildeling af forsøg overordnet den samme uanset fase og terapeutisk område?

Hvilke parametre bedømmer hovedkvarteret jer datterselskaber på forud for og efter et klinisk forsøg?

Outsourcer virksomheden nogen gange beslutninger om lande‐ og site selektion til andre, f.eks. CROer?

Outsourcer I typisk indenfor bestemte faser og/eller bestemte terapeutiske områder?

Er det i så fald både lande‐ og site‐selektion som outsources?

### Del 2

*De næste spørgsmål handler om, hvordan I som virksomhed vurderer et site forud for et eventuelt samarbejde, og hvilke faktorer der er afgørende for, om I vælger at indgå i et sådan samarbejde. Formålet er at belyse, hvilke faktorer I som virksomhed vægter højest, når I vurderer potentielle sites, og på hvilket grundlag vurderingen sker*.

Har I i virksomheden en beslutningsmodel eller fælles retningslinjer, som I benytter ved site selektion?

Hvis ja: Er der fælles retningslinjer for de forskellige datterselskaber?

Hvis nej: På hvilket grundlag sker site selektion så?

Oplever du site selektion‐processen som ensartet fra gang til gang eller som mere ustruktureret?

Er site selektion processen den samme uanset fase og terapeutisk område?

Hvad noterer I jer, når I skal vurdere fordele og ulemper ved et site?

Kan du give eksempler på faktorer, som har stor betydning ved nogle typer forsøg, men mindre betydning ved andre typer forsøg?

Kan du uddybe, hvad I vægter højest?

Er der omstændigheder eller faktorer, som næsten altid spiller en rolle?

Hvis der er tale om et site, I ikke kender fra tidligere, hvilke faktorer lægger I så særlig vægt på?

Hvis du kunne vælge, hvad ville du så helst have, at de sites I arbejder sammen med blev bedre til?

Hvor ofte sker det, at et site som I ønsker at arbejde sammen med i forbindelse med et klinisk forsøg, takker nej til samarbejdet?

Er data fra site vedrørende patientrekrutteringshastighed og ‐succesrate i tidligere forsøg vigtige for jer ved vurderingen af sites? Spørger I systematisk til disse oplysninger ved feasibility eller site selection visit?

Spørger I altid til deviations ved tidligere inspektioner ved site selektion?

Hvor ofte vurderer du, at I i virksomheden vælger at samarbejde med et site, som ikke leverer godt, fordi I skal tage hensyn til specifikke organisationer eller personer så som key opinion leaders?

Vurderer du, at de ting som du har beskrevet, kan siges at være gældende for *hele* virksomheden eller kun den enhed, du sidder i?

Vurderer du, at de andre datterselskaber vurderer sites på samme måde som jer og lægger vægt på de samme faktorer?

Er der yderligere, du vil fremhæve eller tilføje?
